# Literacy Skills, Immigration, and Motivation to Learn Among Middle-Aged and Older Adults in the United States

**DOI:** 10.1093/geroni/igaa057.1059

**Published:** 2020-12-16

**Authors:** Shalini Sahoo, Roberto Millar, Taka Yamashita, Phyllis Cummins

**Affiliations:** 1 University of Maryland, Baltimore, Elkridge, Maryland, United States; 2 Graduate Student, Baltimore, Maryland, United States; 3 University of Maryland, Baltimore, Maryland, United States; 4 Miami University, Oxford, Ohio, United States

## Abstract

Education and training over the life course or lifelong learning has become critical in the fast-changing U.S. society. Foundation skills (e.g., literacy), motivation, curiosity, as well as access to learning opportunities are essential to promote lifelong learning. Despite the importance of these promoting factors, empirical research focusing on complex relationships between literacy skills, immigration and motivation to learn (MtL) among middle-aged and older adults is scarce. The objective of this study is to examine how literacy skills and immigration (vs. U.S. born) are associated with MtL among middle-aged and older adults in the U.S. Nationally representative data (n = 8,670) of adults aged 45 years and older were obtained from the 2012/2014 Program for International Assessment of Adult Competencies (PIAAC). Structural equation models were constructed to examine the formerly tested and validated latent MtL construct based on four 5-point Likert-type scale items among the sub-population of interest. Results showed that higher literacy skills (0-500 points; b = 0.002, p < 0.05) was associated with greater MtL. Additionally, immigrants were less likely (b = -0.114, p < 0.05) to have greater MtL than those who are non-immigrants (i.e. U.S. born). Higher literacy skills may indicate positive experiences in previous adult education and training and greater readiness for further learning. Findings from this study provide new empirical evidence of lifelong learning determinants. Educators and researchers should be aware of limited literacy and being an immigrant as potential barriers to knowledge-seeking in later life.

